# Supplementary material for: Guideline Adherence and Registry Recruitment of Congenital Primary Hypothyroidism: Data from the German Registry for Congenital Hypothyroidism (HypoDok)
Source: Int J Neonatal Screen. 2021 Feb 12;7(1):10. doi: 10.3390/ijns7010010 (PMC8006240; doi:10.3390/ijns7010010)
Supplement: Supplementary file 1 [file IJNS-07-00010-s001.pdf]

Table S1. Statements of the German Guideline for Primary Congenital Hypothyroidism (CH) which were not further examined in the present manuscript [1].

**Statement 5**	• For goiter: sequencing of the TPO gene<br>• With a simultaneous cleft palate: exclusion of FOXE1 gene mutation<br>• For coexistent movement disorders: exclusion of NKX2.1 gene mutation
**Statement 6**	• Sole L-T_4 supplementation is recommended as medication of choice
**Statement 10**	Timing of reevaluation of thyroid function:<br>• In case of iodine exposure: after 3 months of therapy<br>• In case of decreasing previously detected auto-antibody titers or stable blood TSH or if thyroid gland is detectable: after the 2nd year of life

Table S2. Additional data on German newborn screening [2].

Year	Number of confirmed cases of CH in newborn screening	Prevalence	Recall rate [%]	Positive predictive value PPV [%]	Specificity [%]
**2004**	222	1:3,270	0.13	22.67	
**2005**	187	1:3,728	0.24	11.15	99.79
**2006**	165	1:4,156	0.17	14.49	99.86
**2007**	163	1:4,202	0.14	16.98	99.88
**2008**	184	1:3,709	0.09*	28.62*	99.94*
**2009**	195	1:3,441	0.09*	29.39*	99.88*
**2010**	207	1:3,275	0.08*	35.23*	
**2011**	207	1:3,201	0.08*	35.11*	
**2012**	205	1:3,285	0.13		
**2013**	211	1:3,233	0.12		
**2014**	213	1:3,356	0.14		
**2015**	235	1:3,139	0.13		
**2016**	242	1:3,273	0.12		
**2017**	279	1:2,813	0.13	28.01	99.91

*data derived only from tests performed ≥ 36 hours.

Table S3. Morphology of thyroid gland, examined via sonography, divided before and after guideline appearance (groups A and B).

	Group A "before guideline" 10/ 2001 – 1/2011	Group B "after guideline" 2/2011 – 05/2020
**Thyroid gland in loco typico**	61.9 (n=84)	62.7 (n=118)
**Ectopia**	7.4 (n=54)	6.7 (n=90)
**Athyreosis**	24.1 (n=58)	38.5 (n=96)

Results are given in %. N is the total number of patients available for each analysis as explained in the results part of the main article (page 5).

Table S4. Genetic findings indicating a) dysgenesis or b) dyshormonogenesis, c) no mutation found, divided before and after guideline appearance (groups A and B).

	**Group A "before guideline" 10/ 2001 – 1/2011**	**Group B "after guideline" 2/2011 – 05/2020**
	**a) Dysgenesis**	
**NKX2.1/TTF1-gene mutation**	9.1 (n=11)	0.0 (n=13)
**PAX8-gene mutation**	9.1 (n=11)	7.7 (n=13)
**TSH-receptor-gene mutation**	9.1 (n=11)	15.4 (n=13)
	**b) Dyshormonogenesis**	
**SLC26A4 (pendrin)-gene mutation**	18.2 (n=11)	0.0 (n=13)
**Thyroid peroxidase (TPO)-gene mutation**	18.2 (n=11)	38.5 (n=13)
**Thyreoglobuline (Tg) gene mutation**	18.2 (n=11)	0.0 (n=13)
	c) **No mutation found**	
**No mutation found**	18.2 (n=11)	38.5 (n=13)

Results are given in %. N is the total number of patients available for each analysis as explained in the results part of the main article (page 5).

1. H, K. Diagnostik, Therapie und Verlaufskontrolle der Primären angeborenen Hypothyreose. Availabe online: https://www.awmf.org/uploads/tx_szleitlinien/027-017l_S2k_Primaere_Angeborene_Hypothyreose_2011-abgelaufen.pdf (accessed on 30.01.2021).
2. e.V., D.G.f.r.N. DGNS Screeningreports 2004-2017. Availabe online: http://www.screening-dgns.de/reports.php (accessed on 29.01.2021)
